# Scientific Achievements in the Study of the Occurrence and Antimicrobial Susceptibility Profile of Major Foodborne Pathogenic Bacteria in Foods and Food Processing Environments in Romania: Review of the Last Decade

**DOI:** 10.1155/2020/5134764

**Published:** 2020-06-24

**Authors:** Kálmán Imre, Viorel Herman, Adriana Morar

**Affiliations:** ^1^Department of Animal Production and Veterinary Public Health, Faculty of Veterinary Medicine, Banat's University of Agricultural Sciences and Veterinary Medicine “King Michael I of Romania” from Timişoara, 300645, Romania; ^2^Department of Infectious Diseases and Preventive Medicine, Faculty of Veterinary Medicine, Banat's University of Agricultural Sciences and Veterinary Medicine “King Michael I of Romania” from Timişoara, 300645, Romania

## Abstract

Pathogenic bacteria are recognized as a major cause of foodborne diseases in humans, globally, with negative impact on the economy of each country. The aim of the present review was to obtain a comprehensive understanding about the frequency of isolation, diversity, and antimicrobial susceptibility profile of the six major foodborne pathogenic bacteria in food matrices and food processing environment, in Romania. In this regard, results of relevant epidemiological studies, published during the last decade and retrieved from the Web of Science Core Collection database, were analyzed, with special emphasis on scientific achievements, main knowledge gaps, and future perspectives. The summarized and harmonized results offer useful insight, especially for public health authorities and researchers, having a reference effect in stimulating further opportunities for studies to be carried out to address some of the limitations of the current status.

## 1. Introduction

Bacterial foodborne pathogens are considered the most frequently implicated biological agents in food poisoning syndrome in humans, often called foodborne illness (FBI). FBI is usually characterized by acute health problems, with gastrointestinal (e.g., diarrhea, vomiting, nausea, or abdominal cramps), or neurological (e.g., headaches, paralysis, or paresthesia) manifestations. In “*sensu stricto*”, two types of FBI are known, namely infection, which is the consequence of ingesting food or water contaminated with pathogenic bacteria, and intoxication, meaning the situation when the toxin produced by the pathogen causes the illness [1–4].

Currently, due to the existence of several contamination sources with harmful bacteria via the food chain (e.g., animals, soil, water, air, food handlers during production and storage), the obtaining of safe and nutritious food products for the consumer is considered to be a great challenge for the food industry, worldwide. However, adequate cold preservation (e.g., refrigeration or freezing), associated with proper thermal processing of foods can prevent FBI. In case of foodborne diseases, the effectiveness of public health interventions is frequently hampered by the involvement of antibiotic-resistant bacteria. Thus, the continuous monitoring of the antimicrobial resistance phenomenon must be considered a priority for the public health sector [1, 4, 5].

In each year, the European Food Safety Authority (EFSA), together with the European Center for Disease Prevention and Control (ECDC) and with the contribution of each member state, publishes an open access summarized report about the occurrence of foodborne pathogen bacteria and their antimicrobial resistance profile in the human-animal-food chain [1, 4]. Accordingly, year by year, the most frequently registered top five pathogens in descending order, causing severe infections in humans and economic losses are *Campylobacter* spp., *Salmonella* serovars, *Yersinia* spp., shiga toxin-producing *Escherichia coli*, and *Listeria monocytogenes*. The data reported by member states are the result of a harmonized interinstitutional action, which encompasses positive findings from clinical cases, official and national control programs, as well as from the self-control process of the production units [4]. The resulted synopsis from member states, including Romania, does not include results from scientific publications, neither provides exact information about the origin and type of the tested food matrix, in direct association with the detailed antibiotic resistance profile of the implicated bacterial species and/or serotypes. In this regard, summarizing the current understanding, together with the awareness of knowledge gaps in a timely manner, can be the cornerstone of an appropriate control plan of the main bacterial foodborne diseases.

With these considerations, the present review aimed to obtain an overview of the baseline data on the occurrence of major food-borne pathogenic bacteria (*Salmonella* spp., *Listeria monocytogenes*, *E. coli*, *Staphylococcus* spp., *Campylobacter* spp., and *Yersinia* spp.), and their antimicrobial susceptibility profile (where data is available) in a food matrix and/or food processing environment, processing results of relevant epidemiological studies retrieved in the Web of Science Core Collection database and published during the last decade. In the foreground, scientific achievements, main knowledge gaps, and future perspectives are approached.

## 2. Materials and Methods

A search was conducted in the Web of Science Core Collection database, in order to identify papers published in international scientific journals, which have undergone a rigorous peer review process, containing research data generated by Romanian researchers at country-level, between January 1st, 2010 and March 31th, 2020. The search strategy consisted of simultaneous use of three search terms, specifically, the name of one of the targeted foodborne bacteria, including “*Salmonella*”, “*Listeria*”, “*E. coli*”, “*Staphylococcus*” “*Campylobacter*” and “*Yersinia*”, and other two basic search terms namely “Romania” and “food”.

At first, the inclusion criteria were based on the individual screening of the title and abstracts in search of resulting publications. Subsequently, if the basic information was deemed appropriate to meet the goal of the study, it was then included in the review and subjected to further, in-depth analysis.

## 3. Results and Discussion

Initial search in the database generated a total of 61 publications. Notwithstanding of that total, only 21 studies contained data that met our inclusion criteria and were subsequently included and processed in this review. The Table 1 summary presents the harmonized results of the included papers.

### 3.1. *Salmonella* spp.

*Salmonella* spp. is a widely distributed pathogen, causing one of the most feared infections in humans, the so-called salmonellosis. Due to this fact, their presence is closely monitored within the food chain steps. For the finished foodstuffs, the absence of *Salmonella* spp. represents a safety criterion. In agreement with these considerations, the search results revealed that *Salmonella* spp. has been the most studied foodborne pathogen in Romania within the last decade. The processing of the data from the 11 publications dedicated to the *Salmonella* topic has revealed the isolation of a total of 435 strains. Out of them, 402 (92.4%) have been serotyped.

Regarding the investigated food matrices, poultry meat has been reported as being most frequently contaminated with *Salmonella*. Positive results were recorded in both raw chicken meat (118 positive samples from a total of 602) [2–4] and chicken carcass (76/2321) [3, 5, 6] examinations, resulting in an overall prevalence of 19.6% and 3.3%, respectively. Raw pork was another important reservoir for *Salmonella* spp., recording a positivity of 2.2% (151/6887) [2, 4, 6, 7]. In addition to these baseline epidemiological data, in several other studies, the isolation of nine pork [8, 9], and seven chicken [9] origin *Salmonella* strains has been reported, without specifying the total number of processed samples (Table 1). Other studies have revealed that *Salmonella* was identified in 0.1% (2/2499), 0.3% (37/12797), 1.9% (16/833), and 6.3% (3/48) of livestock ruminant carcasses, mechanically processed red meat, mechanically processed poultry meat [6], and shell egg [4] samples, respectively. *Salmonella* spp. were also recorded in 0.1% (4/3172) of different RTE meat products [6] and 8.1% (3/37) of sausages [4]. Contrary, no bacteria were isolated from illegally sold RTE food in Romanian markets [10] and pasteurized mélange [11].

Among the analyzed categories of nonfood matrices, *Salmonella* was recovered from absorbent food pads from packages with raw chicken meat [12], scald water sludge, and detritus from hair removal [7] with different detection rates, as highlighted in Table 1.

Serotyping tools have revealed the occurrence of a total of 35 serotypes. Out of them, *S*. Infantis (29.5%) was the most frequently recorded, with dominant occurrence in chicken meat. In addition, the two most clinically relevant serotypes namely, *S*. Typhimurium (9.3%) and *S*. Enteritidis (6.2%), besides other four, including *S*. Saintpaul (10%), *S*. Bredeney (8.5%), *S*. Ruzizi (6.7%), and *S*. Derby (6.5%), have been frequently recorded circulating among Romanian foods.

Antimicrobial resistance was recorded for at least one of the 37 antimicrobial agents reported as tested. The exhibited resistance profile of the tested *Salmonella* strains varied widely from one study to another (Table 1). The registered differences can be sustained by the use of different testing methodologies and lack of uniformity of the drugs enrolled in the investigations. Nevertheless, the overuse of some antimicrobials (e.g., tetracycline, azithromycin, nalidixic acid, sulfamethoxazole, streptomycin) in Romanian veterinary medicine is noteworthy. Contrary, five studies [3, 4, 7–9] reported complete susceptibility for ceftazidime, which can constitute a useful insight for public health specialists, in the management of human infections.

### 3.2. Listeria Monocytogenes

*L. monocytogenes* is the causative agent of listeriosis in humans, a severe illness with a high fatality rate and atypical evolution. Like in the case of other foodborne diseases, consumers such as young children, elderly people, and pregnant women are more susceptible to foodborne listeriosis than healthy adults [13]. The ready-to-eat (RTE) products are considered high-risk foods, because they support the growth of *L. monocytogenes* and do not require heat treatment before consumption. Also, the persistence of *L. monocytogenes* in the food processing environments is well documented [14]. Therefore, the control of this pathogen for producers of RTE foods is a major challenge.

Over the last decade, in Romania, there was a total of nine studies that published data on the isolation frequency of *L. monocytogenes* from a food matrix or food processing environment. The pathogen was recovered from a variety of food products, including raw (pork, beef, sheep, poultry, and snail) and mechanically processed meat, RTE, milk and dairy products, chicken carcasses, fish, and fresh salads [5, 6, 10, 14–16], with different prevalence values as highlighted in Table 1. Two studies were designed to identify *L. monocytogenes* predominantly in the food processing environment, including food contact and nonfood contact surfaces [14, 17]. These investigations offered baseline information for food safety managers from the surveyed production units, in order to improve their hazard analysis critical control point plans and sanitation programs. Also, the successful isolation of *L. monocytogenes* from household refrigerators draws the attention about the risk that is posed, especially for vulnerable consumers, in case of inadequate cooling practices [13].

Four studies, based on molecular serotyping tools, have provided data on the genetic diversity of a total of 98 L*. monocytogenes* strains [10, 14–16]. The multiplex PCR serotyping techniques have revealed the occurrence of five major subtypes namely, 1/2a (67.3%) as dominant subtype, followed by 1/2c (21.4%), 1/2b (5.1%), 4b (4.1%), and 4a (2.0%), respectively. In one study the multilocus sequence typing highlighted the virulence profile of the isolates, revealing six different sequence types (ST2, ST8, ST9, ST20, ST121, and ST155) (Table 1). Generating such data can be the main epidemiological survey tool in tracking the source of listeriosis cases in human infections.

In the context of the importance of the global fight against antimicrobial resistance, only two studies approaching this problem have been published [5, 17]. The results indicate a high prevalence of multidrug-resistant strains in the pork and poultry meat industries. Encouraging results were obtained regarding the total susceptibility of some tested antimicrobials (Table 1), but caution should be taken in interpreting these results, considering the limited number of the tested *L. monocytogenes* isolates.

### 3.3. Escherichia Coli

Intestinal and extraintestinal pathogenic variants of *E. coli*, which is considered a commensal bacterium of the large intestinal tract of healthy hosts, are able to produce infections in humans and animals, with a wide range of manifestations (e.g., mild to severe diarrhea, hemorrhagic colitis, meningitis, septicemia, hemolytic-uremic syndrome, or urinary tract infections). Thus, according to the pathogenic mechanism and virulence traits, the diarrheagenic *E. coli* strains causing diarrhea syndromes have been classified into six subpathotypes including enterohemorrhagic (EHEC, also known as Shiga toxin—producing *E. coli* [STEC]), enterotoxigenic (ETEC), enteropathogenic (EPEC), enteroaggregative (EAEC), enteroinvasive (EIEC), and attaching and effacing (A/EEC) groups. Human extraintestinal *E. coli* infections are strongly related by the involvement of the uropathogenic *E. coli* (UPEC) strains. As member of the fecal coliform group, *E. coli* is the most commonly used hygienic indicator in the food industry. Presently, within the bacterial genetics' investigations, it is one of the most intensely studied microorganisms, due to its ability to adapt and acquire new genofunds, especially antimicrobial resistance genes [18].

Despite these considerations, a very limited number of studies providing data on the occurrence of *E. coli* strains in different food products have been published over the last decade in Romania. Likewise, three out of five available surveys do not provide differentiation data between pathogenic and nonpathogenic strains [5, 12, 19]. The results published by another study, based on biotyping tools, which investigate a large number of samples [6], tried to cover this gap. Accordingly, only the presence of the biotype I (126/11048, 1.14%) has been demonstrated and none of the verocytotoxin producing *E. coli* O157:H7 (0/454) isolates were identified [6]. Nevertheless, the fact that Romania regularly reports the occurrence of VTEC/STEC infections in humans within the European Centre for Disease Prevention and Control (ECDC) surveillance system [20] opens the opportunity for large scale studies to be carried out, which address some of the limitations of the currently available investigations, with special emphasis on the identification of the O157:H7 *E. coli* serotype within the food chain.

The antimicrobial susceptibility testing profile of a total of 77 chicken meat origin *E. coli* isolates showed variable results, from one study to another [5, 12, 19]. It is noteworthy that the registered high resistance pattern towards some antimicrobials (e.g., tetracycline, ciprofloxacin, sulfamethoxazole) suggests their overuse in the Romanian poultry industry.

### 3.4. Staphylococcus Aureus

*Staphylococcus aureus* is considered one of the main causes of foodborne intoxications, being widespread bacteria throughout nature. Its food poisoning effect is related to the ingestion of the preformed enterotoxin produced by strains developed in a food matrix. Additionally, it is well known that *S. aureus* is able to produce nosocomial and invasive infections in humans (e.g., septicemia, osteomyelitis, skin infections, pneumonia, or infections of the central nervous system) [18, 21].

In Romania, during the last decade, despite the existence of a limited number of studies concerning the occurrence of *S. aureus* within the food chain, the monitoring of this pathogen in illegally sold food products has been attracting increased attention. In this regard, two studies confirmed the occurrence of the methicillin-resistant *S. aureus* (MRSA) strains, reporting positive findings in pork lard samples [21], as well as in different fish, dairy and raw meat products [22] (Table 1). Nowadays, the globally distributed MRSA strains constitute a major concern for the public health, due to their ability to easily gain novel antimicrobial resistance mechanisms [21]. In these studies, successful *Spa* typing of a total of 16 isolates has resulted in the expression of nine profiles, including t304 (3 isolates), t524 (2), t011, t091, t398, t449 (4), t1606 (2), t3625, and t803, respectively. The first five enumerated types have been frequently reported as being implicated in human infections. Other studies have demonstrated the presence of *S. aureus* in drinking milk which resulted in a food poisoning outbreak [23], or its absence in raw and ground meat [6].

The antimicrobial susceptibility profile of the tested *Staphylococcus* strains, presented in two studies [12, 21], shows different resistance levels towards different drugs, and complete susceptibility to others (Table 1), but caution should be taken in drawing valid conclusions, considering the low number of tested samples.

### 3.5. *Campylobacter* spp. and *Yersinia* spp.

From the species belonging to the *Campylobacter* genus, which cause campylobacteriosis, *C. jejuni* and *C. coli* have the most frequent implication. Campylobacteriosis is the most common cause of human bacterial gastroenteritis among European countries, on a yearly basis [24]. Most food poisonings are caused by the consumption of undercooked poultry meat, and the clinical signs include fever, nausea, vomiting, and intestinal disorders.

The review data showed the availability of only three studies. These investigations have been focused on *Campylobacter* monitoring mainly in raw chicken meat [4, 5, 22]. As expected, the overall dominance of *C. jejuni* has been observed, but the isolation of *C. coli* has been also demonstrated in a reduced number of samples (Table 1).

The exhibited multidrug resistance patterns of the isolates highlighted a public health risk, but, at the same time, the total susceptibility against gentamicin, observed in both studies, can constitute an alternative for pathogen control (Table 1). Nonetheless, further investigations, on a larger scale, are still necessary to strengthen this observation.

Human yersiniosis is a foodborne disease, most commonly caused by *Yersinia enterocolitica*, which is a widely distributed pathogen throughout the environment. Despite the fact that yersiniosis is the third most commonly reported bacterial foodborne gastroenteritis within the European Union in the last years, there is no published evidence of the occurrence of *Yersinia* spp. in the Romanian food chain appearing in the database. A single study attempted to fulfill this purpose, but all of the investigated chicken carcass samples were negative.

As has been highlighted in other review reports achieved in the United States [26] and China [27], our summarized data can represent a realistic starting and orientation point for applying small meta-analysis models, individually constructed for pathogen and sample type subgroups, in order to generate hypotheses for future research and risk assessment for food safety [28].

## 4. Conclusion Remarks and Perspectives

Analysis of data obtained from a total of 21 relevant studies showed the common occurrence of the targeted major bacterial pathogens within the food chain, differing broadly in terms of examined matrices and depth of analysis. The resulted overall worrying antimicrobial resistance profile of the tested strains strengthens the urgent need of an integrated surveillance system of the dispersion and transmission of drug-resistant bacteria, within the entire food chain. This action requires an excellent collaboration between the environment, veterinary, and public health sectors, according to the One Health approach.

Although the scientific achievements and progresses are remarkable, it is obvious that the number of published papers is limited, precluding meaningful observations in trends of the pathogen isolation frequency. In addition, the large disparity observed between available investigations, according to the targeted pathogen (e.g., *Salmonella* vs. *Campylobacter* and *Yersinia*), and the lack of homogenous reports between some studies restrict the provision of an accurate and straightforward overview of the available data. These limitations may be related to the existence of routine pathogen surveillance within the self-control process of each production unit in accordance with the EU legislation in force, together with the official controls organized by authorities. In this regard, the highly attractive positive findings that were obtained are not disclosed to the knowledge of experts in order to be compared with research-derived data or integrated in a harmonized preventive risk-based program. Nevertheless, enhancing the involvement of Romanian researchers in international collaborations and successful access to financial support from European or national funding agencies can increase the international visibility of Romanian research in the field of food safety.

## Figures and Tables

**Table 1:** Overview about the occurrence and diversity of major foodborne pathogenic bacteria and their antimicrobial susceptibility profile in foods and food processing environments, in Romania.

No.	Food matrix/environment isolation source (no. positive/examined, %)	Species, serotype (sequence type) (no. of isolates)	Exhibited antimicrobial resistance and susceptibility profile		References
			Resistance (%)	Total susceptibility	
*Salmonella* spp.					
1.	Raw pork (33/146, 22.6)	*S*. Typhimurium (9), *S*. Rissen (8), *S*. Infantis (6), *S*. Bredeney (2), *S*. Derby (2), *S*. Brandenburg (1), *S*. Enteritidis (1), *S*. Gloucester (1), *S*. Goldcoast (1), *S*. Kottbus (1), *S*. Ruzizi (1)	AZM (88.2), TET (54.9), SMX (54.9), CIP (45.1), NAL (43.1), AMP (35.3), CHL (33.3), TGC (25.5), CTX (13.7), CST (13.7), TMP (7.8), GEN (2)	MEM, CAF	Tîrziu et al., [4]
	Raw chicken (12/132, 9.1)	*S*. Infantis (12)			
	Eggshell (3/48, 6.3)	*S*. Enteritidis (2), *S*. Infantis (1)			
	Sausage (3/37, 8.1)	*S*. Typhimurium (2), *S*. Derby (1)			
2.	Absorbent food pads from packages with raw chicken meat (1/24, 4.2)	*S*. Infantis	N. A.		Gaspar et al., [12]
3.	Bovine, sheep carcasses (2/2499, 0.1)	*S*. Manchester	FLU (84.7), CSF (78.5), NAL (78.5), NEO (68.2), SP (65.5), TET (63.9), STR (59.7), NOR (34.0), CXM (30.5), NIT (27.8), CIP (25.7), TIC (23.4), EFX (15.3), OFL (15.0), CAF (12.8), SAM (9.1), GEN (6.9)	—	Zaulet et al., [6]
	Chicken carcasses (34/1888, 1.8)	*S*. Saintpaul (9), *S*. Enteritidis (4), *S*. Typhimurium (4), *S*. Bredeney (3), *S*. Colorado (2), *S*. Derby (2), *S*. Agona (1), *S*. Blockley (1), *S*. Concord (1), *S*. Hadar (1), *S*. Heidelberg (1), *S*. Infantis (1), *S*. Norwich (1), *S*. Reading (1), *S*. Rissen (1), *S*. Tennesse (1)			
	Raw pork (51/6442, 0.8)	*S*. Saintpaul (15), *S*. Bredeney (12), *S*. Enteritidis (6), *S*. Concord (5), *S*. Rissen (4), *S*. Derby (3), *S*. Hadar (3), *S*. Typhimurium (2), *S*. Choleraesuis (1)			
	Mechanically processed poultry meat (16/833, 1.9)	*S*. Saintpaul (4), *S*. Agona (3), *S*. Bredeney (3), *S*. Enteritidis (3), *S*. Concord (1), *S*. Heidelberg (1), *S*. Newport (1)			
	Mechanically processed red meat (37/12797, 0.3)	*S*. Saintpaul (13), *S*. Agona (7), *S.* Typhimurium (9), *S*. Bredeney (2), *S*. Concord (2), *S*. Enteritidis (2), *S*. Hadar (1), *S*. Newport (1)			
	RTE meat products (4/3172, 0.1)	*S*. Hadar (2), *S*. Parkroyal (2)			
4.	Illegally sold RTE food in Romanian markets (0/200)	—	—	—	Ciolacu et al., [10]
5.	Pasteurized melange (0/20)	—	—	—	Nistor et al., [11]
6.	Raw chicken (7/N.A.)	*S*. Infantis	LEV (88.9), NAL (88.9), SMX (88.9), TET (77.8), CIP (55.6), NIT (44.4), AMP (33.3), TMP (33.3), CTX (22.2), AMK (11.1), GEN (11.1), PIP (11.1), TOB (11.1),	CAF	Colobatiu et al., [9]
	Raw pork (2/N.A.)	*S*. Infantis, *S*. Derby			
7.	Chicken carcasses (37/289, 12.8)	*S*. infantis (18), *S*. Bredeney (7), *S*. Virchow (6), *S*. Djugu (4), *S*. Grampian (4), *S*. Brandenburg (1), *S*. Derby (1), *S*. Ruzizi (1)	TET (66.0), NAL (64.3), SMX (64.3), CIP (61.9), STR (59.5), TMP (33.3), AMP (9.5), CHL (7.1), GEN (2.4)	CAF, CTX,	Tîrziu et al., [3]
	Raw retail chicken (5/28, 17.9)				
8.	Chicken carcasses (5/144, 3.5)	*S*. Enteritidis (5)	NAL (40), QUI (40), TET (40), CAF (20), CTX (20), GEN (20), SFN (20), SMX (20), SMX/TMP (20), STR (20)	AMP, CHL, CIP, KAN	Dan et al., [5]
9.	Raw pork (12/47, 25.5), packaged pork products (7/44, 15.9), scald water sludge (1/8, 12.5), detritus from hair removal (6/9, 66.7)	N. A.	TET (61.5), AMP (50), PIP (50), TMP-SMX (34.6), AMC (26.9), NIT (23.1), CFZ (15.4), PIP/TBZ (7.7), CIP (3.8), IMI (3.8), NOR (3.8)	AMK, CAF, CEF, CFX, CTX, GEN	Morar et al., [7]
10.	Raw pork (minced meat) (7/N.A.),	N. A.	AMP (75.0), AMC (62.0), CFZ (62.0), PIP (62.0), TET (50.0), CFX (25), CXM (25.0), CTX (12.0)	AMK, ATM, CAF, CIP, FOS, GEN, IMI, NOR, SMX/TMP	Mateescu et al., [8]
	Sausage (1/N.A.)	*S.* Arizonae			
11.	Raw pork (48/208, 23.1)	*S.* Infantis (73), *S.* Typhimurium (19), *S.* Derby (15), *S.* Colindale (14), *S.* Rissen (6), *S.* Ruzizi (5), *S.* Virkow (5), *S.* Brandenburg (4), *S.* Bredeney (4), *S.* Calabar (1), *S.* Enteritidis (1), *S.* Muenchen (1), *S.* Kortrijk (1).	SMX (87.2%), STR (81.2%), TET (80.5%), NAL (65.10%), CIP (42.9%) AMP (20.8%), CHL (16.9%), CAF (11.4%). GEN (0.7%)	CTX	Mihaiu et al., [2]; Barbour et al., [25]
	Raw chicken (101/442, 22.9)				
*Listeria monocytogenes*					
12.	Domestic refrigerators (2/15, 13.3)	N. A.	N. A.	—	Dumitrașcu et al., [13]
13.	Food contact surfaces (14/60, 23.3)—conveyor belts (5/15, 33); cutting surfaces (1/5, 20); packing surfaces (1/3, 33.3); personnel (1/7, 14.3), processing (3/11, 27.3) and slaughter (3/12, 25) equipment; storage container (0/7)	N.A.	BPN (100), FA (100), IMI (100), FOS (92.0), OXA (92.0), CLI (88.0), RIF (56.0), SMX/TMP (48.0), TET (44.0), CIP (4.0),	ERY, GEN, LNZ, MXF, TPL, VAN, TGC	Sala et al., [17]
	Nonfood contact surfaces (11/37, 29.7)—employee workfow areas (2/8, 25), slaughterhouse drains (5/10, 50), processing plant drains (3/10, 30), cooling chamber walls (1/9, 11.1)				
14.	Different types of meats (7/1841, 0.4)—raw pork (2/292, 0.7), mechanically processed red meat (4/374, 1.1), RTE products (1/1146, 0.1)	N. A.	N. A.	N. A.	Zaulet et al., [6]
15.	Illegally sold RTE foods in Romanian markets (15/200, 7.5)—raw and processed fish (10/61, 16.4), meat and meat products (4/41, 9.8), dairy products (1/73, 1.4)	Genetic lineage I–4b[ST2] Genetic lineage II–1/2a[ST20] (5), 1/2a[ST155] (4), 1/2a[ST8] (2), 1/2a[ST121] (2), 1/2C[ST9]	N. A.	N. A.	Ciolacu et al., 2016; Ciolacu et al., [10]
16.	Nonfood contact surfaces (16/81, 19.8) Food contact surfaces (28/85, 32.9) Raw materials (9/20, 45) RTE meat products (8/40, 20)	42 selected strains: 1/2a[3a] (35), 1/2c [3c] (7)	N. A.	N. A.	Bolocan et al., [14]
17.	Chicken carcasses (19/144, 13.2)	N. A.	TET (31.6), CHL (21.5), QUI (21.1), AMP (10.5), CIP (10.5), NAL (10.5), SFN (5.3), SMX (5.3), SMX/TMP (5.3)	CAF, CTX, GEN, KAN, STR,	Dan et al., [5]
18.	Food products (19/N.A.)—raw minced meat (4), pork (1), beef (1), bacon (2), chicken (2), forcemeat balls (4), sausage (2), shell snails (1), cheese (2)	1/2a[3a] (12), 1/2b[3b] (2), 1/2c[3c] (2), 4a[4c] (2), 4b[4d, 4e] (1)	N. A.	N. A.	Caplan et al., 2014 [16]
19.	Different food products (25/235, 10.6)—unpasteurized milk (3/22, 13.6), sheep cheese (2/15, 13.3), cream (1/18, 5.6), raw sheep (3/25, 12), raw poultry (1/20, 5), raw pork (2/23, 8.7), raw beef (2/20, 10), snail meat (4/30, 13.3), fresh salad (4/15, 26.7), other foods (0/72)	1/2c (11), 1/2a (6), 1/2b (3), 4b (2)	N. A.	N. A.	Carp-Cărare et al., [15]
*E. coli*					
20.	Absorbent food pads from packages with raw chicken meat (8/22, 36.4)	N. A.	CEF (100), FIX (87.5), CIP (75), OFL (75), MEM (12.5)	GEN	Gaspar et al., [12]
21.	Raw meat (1/501, 0.2),	Biotype I (126)	N. A.	N. A.	Zaulet et al., [6]
	Ground meat (125/10548, 1.2)				
22.	Illegally sold RTE foods in Romanian markets (0/200)	O157:H7 (0)	—	—	Ciolacu et al., [22]
23.	Chicken carcasses (18/144, 12.5)	N. A.	TET (66.7), QUI (55.5), NAL (44.4), CIP (33.3), AMP (27.7), CHL (22.2), SFN (22.2), SMX (22.2), SMX/TMP (22.2), STR (11.1), GEN (11.1)	CAF, CTX, KAN	Dan et al., 2015 [5]
24.	Raw chicken (51/100, 51)	N. A.	TET (84.3), AMP (73.0), SMX (66.3), SMX/TMP (48.3), NAL (30.3), CIP (16.8), CHL (14.6), STR (9.0), GEN (7.9), CTX (5.6), CAF (3.4)	—	Colobătiu et al., [19]
*Staphylococcus aureus*/spp.					
25.	Absorbent food pads from packages with raw chicken meat (6/15, 40)	Coagulase positive staphylococci (32)	MET (100), CFX (63.3), SMX/TMP (43.3), OXA (10),	—	Gaspar et al., [12]
26.	Raw and ground meat (0/212)	N. A.	N. A.	N. A.	Zaulet et al., [6]
27.	Illegally sold RTE foods in Romanian markets (16/200, 8)—raw and processed fish (5/61, 8), meat and meat products (3/41, 7.3), dairy products (8/72, 11.1), other products (0/16)	*S. aureus* (16)—t449 (4), t304 (3), t1606 (2), t524 (2), t011, t091, t3625, t803, unknown (1)	N. A.	N. A.	Ciolacu et al., [22]
		*S. aureus*—MSSA/MRSA (9), MRSA (5), MSSA (1)			
28.	Black-market sold food (16/200, 8)	*S. aureus*—MSSA (31), MRSA—t398 (1)	PEN (31.3), TET (12.5), CIP (6.3), AMC (3.1), CFZ (3.1), OXA (3.1)	AMK, CLI, DAP, ERY, FA, FOS, GEN, LEV, LNZ, MUP, RIF, TOB, TPL, VAN	Oniciuc et al., [21]
29.	Milk (2/2, 100)	*S. aureus*—t902 (2)	N. A.	N. A.	Coldea et al., [23]
*Campylobacter* spp.					
30.	Raw chicken (10/34, 29.4)	*C. coli* (7), *C. jejuni* (3)	CIP (80), NAL (80), TET (40), ERY (10), STR (10)	GEN	Tîrziu et al., [4]
31.	Illegally sold RTE foods in Romanian markets (0/200)	—	—	—	Ciolacu et al., [22]
32.	Chicken carcasses (22/144, 15.3)	*C. jejuni* (22)	TET (31.8), SFN (13.6), SMX (13.6), SMX/TMP (13.6), CHL (9.1), CIP (9.1), NAL (9.1), QUI (9.1), AMP (4.5),	GEN, CTX, CAF, KAN, STR,	Dan et al., [5]
*Yersinia* spp.					
33.	Chicken carcasses (0/144)	*—*	—	—	Dan et al., [5]

Legend: AMC: amoxicillin-clavulanic acid; AMK: amikacin; AMP: ampicillin; ATM: atromycin; AZM: azithromycin; BPN: benzylpenicillin; CAF: ceftazidime; CEF: cefepime; CFX: cefoxitin; CFZ: cefazolin; CHL: chloramphenicol; CIP: ciprofloxacin; CLI: clindamycin; CSF: colistinsulfate; CST: colistin; CTR: cotrimoxazole; CTX: cefotaxime; CXM: cefuroxime; DAP: daptomycin; EFX: enrofloxacin; ERY: erythromycin; FA: fusidic acid; FIX: cefixime; FLU: flumequine; FOS: fosfomycin; GEN: gentamicin; IMI: imipenem; KAN: kanamycin; LEV: levofloxacin; LNZ: linezolid; MEM: meropenem; MET: methicillin; MRSA: methicillin-resistant *S. aureus*; MSSA: methicillin-sensitive *S. aureus*; MUP: mupirocin; MXF: moxifloxacin; NAL: nalidixic acid; NEO: neomycin; NIT: nitrofurantoin; NOR: norfloxacin; OFL: ofloxacin; OXA: oxacillin; PEN: penicillin; PIP: piperacillin; QUI: quinolones; RIF: rifampicin; SAM: ampicillin-sulbactam; SFN: sulfonamides; SMX: sulfamethoxazole; SP: spectinomycin; STR: streptomycin; TBZ: tazobactam; TET: tetracycline; TGC: tigecycline; TIC: ticarcillin; TMP: trimethoprim; TOB: tobramicin; TPL: teicoplanin; VAN: vancomycin; N.A. : not available data.
